# Examining perceptions of the usefulness and usability of a mobile-based system for pharmacogenomics clinical decision support: a mixed methods study

**DOI:** 10.7717/peerj.1671

**Published:** 2016-02-08

**Authors:** Kathrin Blagec, Katrina M. Romagnoli, Richard D. Boyce, Matthias Samwald

**Affiliations:** 1 Center for Medical Statistics, Informatics, and Intelligent Systems, Medical University of Vienna, Vienna, Austria; 2 Department of Biomedical Informatics, University of Pittsburgh, Pittsburgh, Pennsylvania, United States

**Keywords:** Pharmacogenetics, Individualized medicine, Decision support systems, User studies, Clinical, Mixed methods studies

## Abstract

**Background.** Pharmacogenomic testing has the potential to improve the safety and efficacy of pharmacotherapy, but clinical application of pharmacogenetic knowledge has remained uncommon. Clinical Decision Support (CDS) systems could help overcome some of the barriers to clinical implementation. The aim of this study was to evaluate the perception and usability of a web- and mobile-enabled CDS system for pharmacogenetics-guided drug therapy–the Medication Safety Code (MSC) system–among potential users (i.e., physicians and pharmacists). Furthermore, this study sought to collect data on the practicability and comprehensibility of potential layouts of a proposed personalized pocket card that is intended to not only contain the machine-readable data for use with the MSC system but also human-readable data on the patient’s pharmacogenomic profile. **Methods.** We deployed an emergent mixed methods design encompassing (1) qualitative interviews with pharmacists and pharmacy students, (2) a survey among pharmacogenomics experts that included both qualitative and quantitative elements and (3) a quantitative survey among physicians and pharmacists. The interviews followed a semi-structured guide including a hypothetical patient scenario that had to be solved by using the MSC system. The survey among pharmacogenomics experts focused on what information should be printed on the card and how this information should be arranged. Furthermore, the MSC system was evaluated based on two hypothetical patient scenarios and four follow-up questions on the perceived usability. The second survey assessed physicians’ and pharmacists’ attitude towards the MSC system. **Results.** In total, 101 physicians, pharmacists and PGx experts coming from various relevant fields evaluated the MSC system. Overall, the reaction to the MSC system was positive across all investigated parameters and among all user groups. The majority of participants were able to solve the patient scenarios based on the recommendations displayed on the MSC interface. A frequent request among participants was to provide specific listings of alternative drugs and concrete dosage instructions. Negligence of other patient-specific factors for choosing the right treatment such as renal function and co-medication was a common concern related to the MSC system, while data privacy and cost-benefit considerations emerged as the participants’ major concerns regarding pharmacogenetic testing in general. The results of the card layout evaluation indicate that a gene-centered and tabulated presentation of the patient’s pharmacogenomic profile is helpful and well-accepted. **Conclusions.** We found that the MSC system was well-received among the physicians and pharmacists included in this study. A personalized pocket card that lists a patient’s metabolizer status along with critically affected drugs can alert physicians and pharmacists to the availability of essential therapy modifications.

## Introduction

In the past two decades, Pharmacogenomics (PGx) has become a promising area in the field of personalized medicine. There is a growing body of literature that emphasizes the influence of genetic variants on the rate of adverse drug events or drug inefficacy and several working groups have been formed with the aim of developing and publishing pharmacogenetics-based drug dosing guidelines (Caudle et al., 2014; Swen et al., 2011; Pirmohamed et al., 2013). Nevertheless, clinical application of pharmacogenetic knowledge has been slow and is largely reserved to specialized centers and clinical trials (Hoffman et al., 2014; Gottesman et al., 2013; O’Donnell et al., 2014; Pulley et al., 2012). One of the reasons for this might be physicians’ lack of education in pharmacogenetics and consequently lack of confidence in dealing with such information (Haga et al., 2012).

Pharmacogenomic Clinical Decision Support (CDS) systems, if well-designed, could help to overcome these challenges. CDS systems are computer based systems that are developed to assist and improve medical decision making at the point of care by providing health care providers with intelligently filtered knowledge, for example patient-specific treatment recommendations. CDS systems can either be integrated into existing health IT infrastructure, such as the Electronic Health Record (EHR) and Computerized Provider Order Entry (CPOE), or they can be designed as separate programs, web services or mobile applications. Furthermore, CDS systems can be distinguished by the way the user is presented with relevant information: In active CDS, an automatic alert is triggered in response to an event or action, e.g. a certain drug is prescribed to a patient who exhibits risk factors that make him more vulnerable to developing adverse drug reactions. In contrast, passive CDS requires the user to actively seek out the recommendation, for example, by clicking on a button or by opening a tab. Studies suggest that physicians have a positive attitude towards CDS systems, appreciating them as tools to manage and make optimal use of the large amounts of complex information that they are often confronted with (Varonen, Kortteisto & Kaila, 2008; Zaidi, Marriott & Nation, 2008). Also, there is evidence that the implementation of CDS systems can positively influence health care processes (Jaspers et al., 2011; Bright et al., 2012). Finally, the amount of PGx knowledge available in structured formats that can be utilized for CDS is improving (Boyce et al., 2013). However, clinical implementation of CDS systems is often hindered by usability issues, lack of user acceptance and uncertainty on how to integrate such systems efficiently into existing and diverse workflows (Kawamoto, 2005). These issues are especially salient in settings where sophisticated CDS systems with the ability of actively generating PGx-based alerts during order entry are lacking.

In recent years, a considerable amount of literature has been published on the development and implementation of PGx CDS systems (Goldspiel et al., 2014; Hicks et al., 2012; Rasmussen-Torvik et al., 2014; Devine et al., 2014; Overby et al., 2012). However, most of them were specifically tailored for use within the local EHR or CPOE, and thus their application is restricted to the respective local health IT infrastructure. In contrast, few approaches exist to make PGx CDS independent of the local infrastructure and IT capabilities. Examples of such efforts include a freely available web-based platform that helps estimating a patient’s therapeutic warfarin dose based on PGx variants and other clinical parameters, such as body weight and co-medication (www.WarfarinDosing.org). Other mobile solutions for genomics CDS include the Genomics Advisor app which focuses on predicting disease risk for diabetes and associated comorbidities (Alterovitz et al., 2015).

The purpose of this study is to evaluate the perception of potential users (i.e., physicians and pharmacists) on the usefulness and usability of a flexible mobile-based CDS system for pharmacogenetics-guided drug therapy that can be easily integrated into existing care processes and infrastructures: the Medication Safety Code (MSC) system. The MSC system makes it possible to store PGx patient data in compact two-dimensional Quick Response (QR) codes which can be decoded and interpreted by common smartphones and other devices. The QR code can be included in paper-based lab reports or can be printed on personalized cards. Patients can carry these cards in their wallets and display them to medical professionals when pharmacotherapy is initiated or altered. After scanning the QR code, the medical professional is led to a website that provides decision support messages customized to the PGx profile of the patient. The website provides information on all drugs for which clinically significant and actionable PGx findings are available, placing drugs for which the patient’s specific genetic profile would indicate a deviation from standard therapy on top. Links below each recommendation allow medical professionals to explore full guideline texts and original references at the Pharmacogenomics Knowledgebase (PharmGKB) website. A screenshot of the MSC interface is shown in Fig. 2. A typical MSC QR code that fits on a credit-card-sized personalized card has the capacity to store PGx test results (i.e. haplotypes and phenotypes) for 20 genes. Generating a MSC that stores PGx data on more than 20 genes is possible, but would require a less compact size of the QR code. More detailed descriptions of the Medication Safety Code service and the underlying technology are available from previous publications (Samwald & Adlassnig, 2013; Miñarro-Giménez et al., 2014).

The goal of this study was to (1) evaluate the perception and usability of the MSC system among potential users (i.e. physicians and pharmacists) and to (2) collect data on the practicability and comprehensibility of potential layouts of the proposed personalized pocket card.

## Methods

### Study design

**Figure 1:**
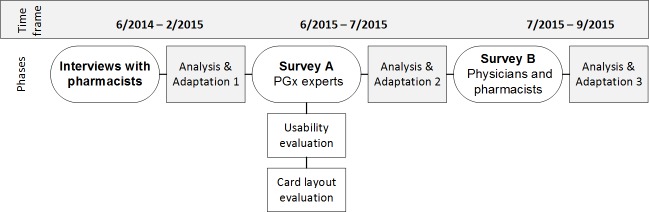
Study design.

We chose an emergent mixed method design encompassing qualitative interviews and quantitative surveys with qualitative elements to allow for a deeper insight into the perception and usability of the MSC system among potential user groups. A multi-method approach was adopted because of its complementary effect in detecting potential usability issues and other barriers that might hinder the implementation of such a system (Walji et al., 2014). The study was conducted in three consecutive phases between June 2014 and September 2015 that built on one another: (1) An initial qualitative interview study among pharmacists and pharmacy students, (2) a web-based survey among PGx experts coming from a wide range of relevant disciplines (“Survey A”), (3) a web-based survey among physicians and pharmacists (“Survey B”). After each stage, an analysis and interpretation of the collected data was performed, followed by an adaptation phase in which the results were used to inform the further development and evaluation process of the MSC system (see Fig. 1).

### Medication Safety Code system

#### User interface

**Figure 2:**
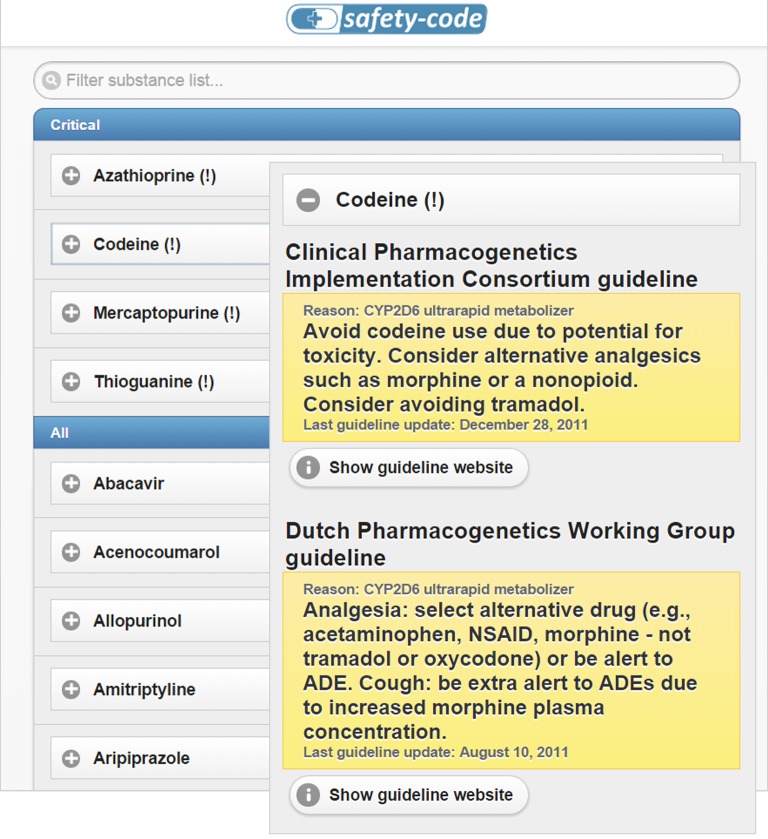
Two screenshots of the MSC interface. Patient-specific guidelines for codeine are shown for a hypothetical patient who has pharmacogenetic test results available that identify him as “CYP2D6 ultrarapid metabolizer” and “TPMT poor metabolizer.” The screenshots depict the version of the user interface that was used during the pilot interviews with pharmacists.

A demo version of the MSC decision support system for a fictional patient (CYP2D6 ultrarapid metabolizer, TPMT poor metabolizer) was used for each of the interviews conducted during the study and for both web-based surveys. In the interviews, participants were presented with a patient scenario in which codeine was prescribed to a fictional patient named “Marilyn” (CYP2D6 ultrarapid metabolizer). Individuals that are CYP2D6 ultrarapid metabolizers have additional copies of the CYP2D6 gene leading to an increased metabolism of several drug substances and consequently a higher chance of adverse drug reactions or drug inefficacy. For codeine, the CYP2D6 ultrarapid metabolizer phenotype is associated with higher rates of adverse drug events and therefore guidelines suggest to avoid prescribing codeine to individuals with this phenotype. For both online surveys, two hypothetical patient scenarios were presented (prescription of codeine for a CYP2D6 ultrarapid metabolizer and prescription of azathioprine for a TPMT poor metabolizer; see Table 1). Patients that are TPMT poor metabolizers are at higher risk of developing life-threatening myelosuppression when treated with standard doses of thiopurine drugs such as azathioprine, 6-mercaptopurine or 6-thioguanine. In total, the system thus highlighted critical recommendations for four drugs, i.e., codeine, azathioprine, mercaptopurine and thioguanine (Fig. 2). Within the first adaptation phase, the MSC UI was split into two different versions to avoid confusion about displaying two different guidelines for the same drug: a “U.S. version” displaying the CPIC guidelines and a “European” version displaying the DPWG guidelines (see Table 6).

**Table 1:** Patient scenarios used for the MSC evaluation.

**Patient scenario 1**
A 35-year-old patient suffering from severe, steroid-refractory Crohn’s disease with extraintestinal manifestations is to be treated with azathioprine. He has pharmacogenomic test results available, identifying him to be a TPMT poor metabolizer. Solely based on these test results and the recommendations provided by the MSC (regardless of other factors such as renal function or drug interactions), what would you recommend for this patient? (more than one answer possible; TPMT: thiopurine S-methyltransferase, an enzyme)
Prescribe azathioprine at normal dosage Prescribe azathioprine at reduced dosage Prescribe a different drug substance
**Patient scenario 2**
A 19-year-old patient suffering from post-operative pain is to be treated with codeine. She has pharmacogenomic test results available, identifying her to be a CYP2D6 ultrarapid metabolizer. Solely based on these test results and the recommendations provided by the MSC (regardless of other factors such as renal function or drug interactions), what would you recommend for this patient? (more than one answer possible)
Prescribe codeine at normal dosage Prescribe a different drug substance, e.g. morphine Prescribe a different drug substance, e.g. tramadol

#### Pocket card layouts

**Figure 3:**
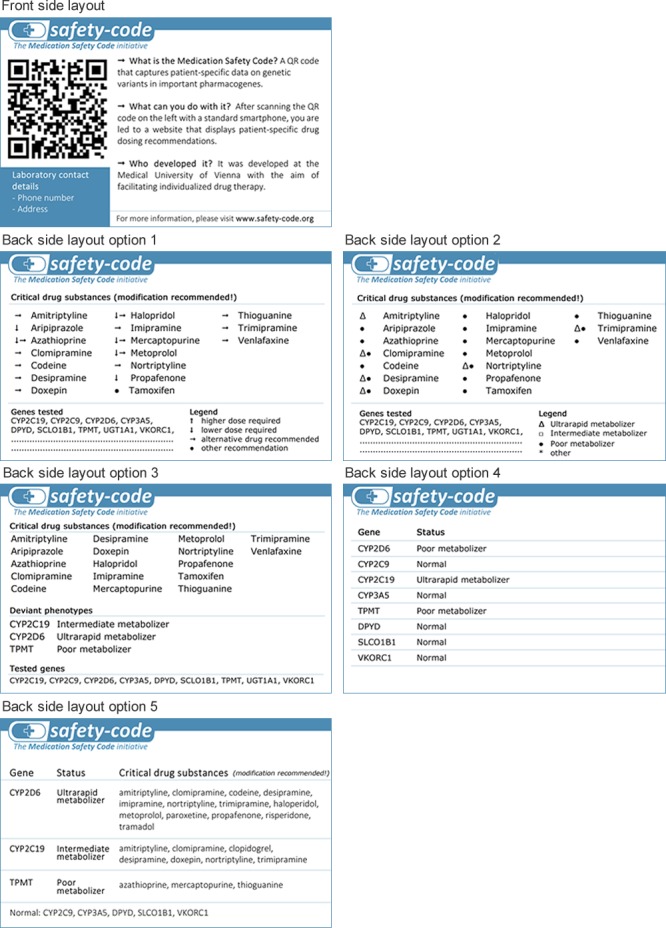
Card layout mock-ups. The front side contains the QR code and general information. The back side is intended to contain a summary of the patient’s pharmacogenomic profile to allow for a quick decision if it is worth to scan the QR code.

Besides the QR code and some personal information (i.e., patient name, date of birth, card number, issue date, name of the providing laboratory), the pocket card is intended to contain human-readable information on the patient’s PGx profile to quickly determine if relevant, actionable genetic variants are actually present. For this study, five different mock-ups of potential card layouts were created to facilitate the visualization of how such information could be represented on the card (see Fig. 3).

### Interviews

#### Data collection

The interviews were conducted as part of a larger-scale study carried out by KR that focused on pharmacists’ general need of PGx information. This study was carried out in an academic health system in Western Pennsylvania and an associated private nursing home prescription benefits management organization. Out of the original sample of 14, a convenience sample of eight participants (five clinical pharmacists and three pharmacy students) were recruited for the MSC usability study and interviewed. The study was classified as exempt by the University of Pittsburgh institutional review board.

An information leaflet listing key facts about the MSC system was given to each participant at the outset of the interview (see Additional file 1). Each interviewee was presented with a patient scenario in which codeine was prescribed to the fictional patient named “Marilyn.” They were then asked to make a recommendation based on Marilyn’s genetic profile (CYP2D6 ultrarapid metabolizer) and the pharmacogenetic decision support messages displayed in the MSC user interface.

The second part of the interview followed a semi-structured interview guide to explore the participants’ general perception of the MSC system and its appearance, their concerns about the MSC system, potential barriers to incorporation of such a system into their workflows, and questions about whether the MSC provides sufficient information to make them feel confident in giving a recommendation (see Additional file 2).

#### Data analysis

Interviews were audio-recorded, transcribed verbatim and analyzed for themes. The length of the interviews ranged between four and fourteen minutes with a mean length of approximately ten minutes. The transcripts were qualitatively coded by one researcher based on the themes covered by the interview guide. In a second step, categories were inductively split up further into specific sub-categories to identify recurrent themes in the participants’ answers. Results were discussed among co-authors.

### Web-based surveys

#### Design

We conducted two separate online surveys aiming at two different target groups to capture a breadth of viewpoints: Survey A was addressed at PGx experts from various disciplines while Survey B specifically focused on end users (i.e., physicians and pharmacists) without particular expertise in PGx. The decision of splitting the surveys and restricting the target group of Survey A to PGx-experienced professionals instead of conducting one all-encompassing survey was based on the assumption that sound PGx knowledge is indispensable for giving informed feedback on how and what human-readable PGx information should be represented on the pocket card. The main aim of Survey A therefore was to get feedback from PGx-experienced professionals on potential layouts of the MSC pocket card. For this purpose, five layout mock-ups that had to be rated from 1 (not practical) to 5 (very practical) were presented to the participants. Additionally, this part of the questionnaire contained 4 multiple choice questions and free text fields asking the participants which information should be printed on the card and how this information should be presented.

In the second part of Survey A, the MSC user interface was evaluated based on two hypothetical use cases and six follow-up questions including free-text fields.

Survey B, on the other hand, focused on evaluating physicians’ and pharmacists’ attitudes towards the MSC system based on two hypothetical patient scenarios and 25 follow-up questions including a 16-item MSC evaluation Likert scale encompassing the following four subscales: (1) usability, (2) trustworthiness, (3) usefulness, (4) workflow integration. This scale was based on the System Usability Scale (SUS) (Brooke, 1996) but extended based on the results of the preceding interview study and our specific target group (e.g., we extended the scale with 4 items regarding workflow integration which was identified as a probable implementation barrier in the preceding interviews). Furthermore, participants’ awareness of and experience with PGx and CDS systems were assessed.

The patient scenarios used for both surveys are shown in Table 1. In both surveys, participants could choose if they want to test the “European” (displaying the DPWG guidelines) or the “U.S.” version (displaying the CPIC guidelines). The questions on the patient scenarios were clearly answerable with both versions. Furthermore, participants could choose between accessing the demo site by either scanning the QR code on the screen or clicking on a link. Both questionnaires also contained a demographics section that included questions regarding participants’ field of work and work experience.

Prior to data collection, the surveys were pre-tested by 4 individuals with medical background to determine and eliminate any weaknesses and ambiguities of the questionnaire. Overall, 16 issues and suggestions for improvements were identified during the pre-test phase and taken into account for revising the questionnaires. Ethical approval for both surveys was obtained from the ethics committee of the Medical University of Vienna (No. 1417/2015).

#### Data collection

For Survey A, PGx-experienced professionals were recruited via e-mail through personal contacts and by distribution via the AMIA Genomics and Translational Bioinformatics (Gen-TBI) Working Group and the PharmGKB network. For Survey B, physicians and pharmacists were recruited via e-mail invitations and through advertisements in professional networks. The first 10 respondents of Survey A and the first 40 respondents of Survey B were eligible for a $17/15€ Amazon voucher.

#### Data analysis

The card layout ratings of Survey A were analyzed descriptively and tested for statistically significant differences using Wilcoxon signed-rank tests. For the MSC evaluation instrument, summing up the subscale scores formed the instruments’ total score. Basic descriptive statistical measures were calculated for each MSC evaluation subscale and for the total scale. Reliability of the subscales was calculated using Cronbach’s alpha (Cronbach, 1951). Besides a descriptive analysis of the evaluation results, non-parametric tests (Mann Whitney U-test and Kruskal Wallis test) were used to test for statistically significant differences in the total MSC evaluation score based on profession, awareness of and experience with CDS systems and genome-guided prescribing. Furthermore, a content analysis of the text box questions, such as comment fields, was conducted for both surveys. Statistical analyses were performed using SPSS 20.

### Demographics

#### Interviewed pharmacists

All five pharmacists were female and aged between 36 and 50. Two of them had been working as pharmacists between five and 15 years, another two between 16 and 25 years and the remaining one stated having less than five years of work-experience. The participants were either working as clinical consultants in nursing homes (n = 3), clinical pharmacists in family practice (n = 1) or as out-patient pharmacists/director of pharmacy residency and advanced practice (n = 1). Out of the five working pharmacists, two stated that they had already been professionally exposed to PGx. However, none of them had ever been in contact with patients that had pharmacogenetic test results available. Furthermore, none of them had ever recommended pharmacogenetic testing to a patient so far. The three interviewed students were aged between 18 and 35, two were male and one was female. All of them stated that they had learned about the application of pharmacogenetics in pharmacy school didactic coursework and had participated in research involving the presentation of pharmacogenetic information. None of them ever had previous contact with patients with pharmacogenetic test results.

#### PGx experts in Survey A

By the end of the survey period, data had been collected from 63 individuals. Out of those, 9 respondents were excluded because they did not match the target group of “PGx experts” (e.g., participants who stated being pharmacy or medical students were excluded). Out of the remaining 54 respondents, 44.4% were female (see Table 2). Physicians and pharmacists accounted for 33.3% and 31.4% of the participants, respectively. The remaining respondents consisted of researchers (22.2%) and PGx experts from other disciplines (e.g., software developers) (13%). The majority of respondents (90.7%) were US residents, 5.6% were European residents and the remaining 3.7% were located in other regions of the world (i.e., New Zealand and Egypt).

**Table 2:** Participant demographics of Survey A and B. The participant demographics of the interviewed pharmacists and pharmacy students (n = 8) are described in the text.

	Survey A		Survey B	
**Gender**	n	%	n	%
Female	24	44.4	15	38.5
Male	30	56.6	24	61.5
**Age**	n	%	n	%
20–29	10	18.5	17	43.6
30–39	24	44.4	12	30.8
40–49	10	18.5	4	10.3
50–59	7	13.0	4	10.3
60 or older	3	5.6	2	5.1
**Profession**	n	%	n	%
Pharmacist	18	33.3	11	28.2
Physician	17	31.5	28	71.8
Clinician at hospital	15	27.8	4	10.3
Doctor-in-training	–	–	12	30.8
Resident doctor	1	1.9	11	28.2
Other	1	1.9	1	2.6
Researcher	12	22.2	–	–
Other	7	13.0	–	–
**Country**	n	%	n	%
USA	49	90.7	–	–
Austria	–	–	17	43.6
Germany	–	–	18	46.2
Other	5	9.3	4	10.2
**Years in work field**	n	%	n	%
>20 years	8	14.8	4	10.3
11–20 years	13	24.1	3	7.7
6–10 years	14	25.9	6	15.4
0–5 years	19	35.2	26	66.6
**Total**	54	100	39	100

#### Physicians and pharmacists in Survey B

A total of 450 physicians and pharmacists were invited via e-mail. Out of those invitations, 28 were undeliverable. Twenty-six of the invited individuals completed the questionnaire (response rate 6.2%). Advertisements in professional networks of the co-authors accounted for 17 additional respondents, resulting in a total number of 43 participants. Of these 43 participants, 4 had to be excluded. Out of the remaining 39 respondents, 11 (28.2%) were pharmacists (see Table 2). The overwhelming majority of participants were from Austria and Germany (43.6% and 46.2%, respectively). 56.4% and 69.2% of the participants stated that they were aware of genome-guided prescribing and CDS systems, respectively.

Over half of the respondents indicated that they were at least sometimes using CDS systems (56.4% sometimes and 5.1% often). In contrast, only 30.8% of the participants were sometimes performing genome-guided prescribing, whereas the remaining 69.2% were never performing genome-guided prescribing.

## Results

### Evaluation of the MSC system

#### Patient scenarios

All of the eight interviewees came to the conclusion to avoid prescribing codeine to the fictional patient Marilyn due to her CYP2D6 ultrarapid metabolizer phenotype. Two out of the eight participants named a specific drug (i.e., Tylenol [active ingredient: Acetaminophen] and Morphine) which they would recommend to prescribe as an alternative to codeine to Marilyn based on the guideline text. Six interviewees formed their conclusions solely based on the information displayed by the MSC user interface. The remaining two participants would have preferred to additionally visit the references prior to making their final recommendation. However, this was not possible in the controlled study setting. Likewise, almost all of the respondents of Survey A and B decided to treat patient 1 in accordance with the DPWG or CPIC guidelines. Interestingly, 12.7% of the PGx experts decided to prescribe azathioprine at normal dosage regardless of the patient’s TPMT poor metabolizer phenotype. Among the participants of Survey B only 2 individuals (4.3%) would have prescribed the standard dosage of azathioprine (see Table 3). In patient scenario 2, 72.7% of the PGx experts and 64.1% of the physicians and pharmacists of Survey B would have acted in accordance with the guidelines.

**Table 3:** Results of the patient scenarios in Survey A (PGx experts) and Survey B (physicians and pharmacists). Recommended treatments according to the DPWG and CPIC guidelines are marked with asterisks.

	Survey A	Survey B
**Patient scenario 1**	**n (%)**	**n (%)**
*Prescribe azathioprine at reduced dosage	32 (45.1)	20 (42.6)
*Prescribe a different drug substance	30 (42.3)	25 (53.2)
Prescribe azathioprine at normal dosage	9 (12.7)	2 (4.3)
**Patient scenario 2**	**n (%)**	**n (%)**
*Prescribe a different drug substance, e.g. morphine	40 (72.7)	25 (64.1)
Prescribe codeine at normal dosage	8 (14.6)	5 (12.8)
Prescribe a different drug substance, e.g. tramadol	7 (12.7)	9 (23.1)

#### General perception

The interviewees’ reaction to the MSC user interface was consistently positive. Three-quarters of the interviewed participants explicitly mentioned the appearance (i.e., layout, formatting and drug categorization) of the MSC interface as appealing. Likewise, across both surveys the majority of participants agreed (57.6%) or strongly agreed (9.8%) that the MSC UI design was appealing. Half of the interviewed pharmacists stated that they appreciated the conciseness and ease of use of the MSC interface. Other themes that were perceived positively by at least two of the interviewed pharmacists were the links to the references and the fact that a recommendation for an alternative drug was stated in the guideline text for codeine. Two of the interviewed pharmacists liked the fact that guidelines by two different groups were provided by the MSC, while on the other hand three participants considered this as negative and confusing.

“For codeine both of the guidelines are saying that there is a problem but if there is not an agreement on that, what would I consult next?”–Pharmacy student 1.

Almost all of the interviewees showed great interest in visiting the references (i.e., PharmGKB website) by clicking on the “Show guideline website” button. Across both surveys, half of the overall participants (50.5%) stated that they had also explored the PharmGKB website.

Table 4 presents the descriptive statistical measures for each subscale and in total for the MSC evaluation among physicians and pharmacists in Survey B. On average, the participating physicians rated the MSC system higher, but not significantly higher than the participating pharmacists (see Table 5). Awareness of and prior experience with CDS systems and genome-guided prescribing did not significantly influence the participants attitude towards the MSC system. 46.2% of the respondents tested the MSC system by scanning the QR code that led to the demo website. Over half of the participating physicians and pharmacists confirmed that they would appreciate the availability of an additional booklet containing all relevant PGx recommendations to be able to look them up without having to use a computer or mobile phone.

**Table 4:** Descriptive statistical parameters and Cronbach’s alpha for the MSC evaluation subscales and total scale. Higher scores represent more positive responses. All items were 5-point (0–4) Likert items. The maximum scores for each subscale and in total were 16 and 64, respectively.

Scale	# Items	n	Median	IQR	Mean	SD	Alpha
Usability	4	39	11	5.0	10.6	3.1	0.8
Trustworthiness	4	39	10	4.0	10.5	2.4	0.7
Usefulness	4	39	12	3.0	11.4	2.1	0.7
Workflow integration	4	39	10	4.0	9.9	2.3	0.5
**Total scale**	**16**	**39**	**42**	**11**	**42.3**	**8.1**	**0.9**

**Table 5:** Comparison of scores for the total scale between different subgroups of respondents. Higher scores represent more positive responses.

	MSC total score		
Independent variable	Mean (SD)	Median (IQR)	p-value
**Awareness of GGP**			
Aware	41.3 (9.2)	41.0 (13)	0.069 (NS)^a^
Unaware	43.7 (6.5)	45.5 (7)	
**Awareness of CDSS**			
Aware	41.5 (9.1)	41.0 (13)	0.133 (NS)^a^
Unaware	44.2 (7)	45.5 (7)	
**Use of GGP**			
Never	43.0 (7.2)	45.0 (8)	0.142 (NS)^a^
Sometimes	40.8 (10.1)	37.0 (7)	
Often	–	–	
**Use of CDSS**			
Never	45.1 (9.2)	46.0 (13)	0.215 (NS)^b^
Sometimes	40.4 (7.3)	40.0 (11)	
Often	43.5 (2.1)	43.5 (–)	
**Occupation**			
Physicians	43.7 (8.6)	42.5 (11)	0.089 (NS)^a^
Pharmacists	38.8 (5.5)	37.0 (10)	

**Notes:**

Maximun score: 64.

a Mann Whitney U-Test.

b Kruskal Wallis Test.

GGP, genome-guided prescribing; CDSS, clinical decision support systems; NS, not statistically significant.

Statistical significance at 0.05.

#### Workflow integration and balance between too little information and information overload

Seven out of the eight interviewees stated that the MSC system would fit well into their workflow, one participant expressed concerns regarding time as the limiting factor for a successful incorporation. Lack of time was also seen as a probable barrier by two others of the interviewed pharmacists and pharmacy students.

“Probably time, if it’s really busy at the pharmacy this might be something that’s overlooked, especially if it’s not mandatory, the pharmacist has to do it then it just might be overlooked.”–Pharmacy student 2.

Among the participating physicians and pharmacists of Survey B, the median score for the workflow integration subscale was 10 (maximum score 16, see Table 4). Regarding integration into workflow, a common wish amongst the interviewed pharmacists was the integration of the information provided by the MSC into the patient chart after scanning the QR code once or incorporation into the electronic health record/Computerized Provider Order Entry (CPOE) system.

“I need it to be in the chart, because the next time, I don’t want to have to look it up every single time”–Clinical pharmacist 4.

Three-quarters of the interviewed pharmacists and pharmacy students agreed that the MSC system provided enough information for making them feel comfortable in giving a recommendation about a drug/phenotype combination. The median score for the trustworthiness scale in Survey B was 10 (see Table 4). One of the interviewed pharmacists said that they would prefer to have more background information in case they are asked by a physician. However, two interviewees felt that the amount of text might already be too large to read for a physician/pharmacist in a busy setting and one of them suggested the highlighting of keywords to resolve this issue. One participant indicated that the guidelines displayed by the MSC make them confident to recommend avoiding codeine but not in terms of alternative drugs. A concrete listing of alternative therapy options was also something one quarter of the interviewees found lacking on the MSC interfaces. Likewise, one PGx experienced physician commented on his choice in the first patient scenario in Survey A as follows:

“Would also follow the recommended dosing interval and monitoring instructions. I would potentially use an alternative medication, but the alert doesn’t tell me what alternatives would be appropriate (nor did the guidelines website).”

Three interviewees indicated that they would not only appreciate a listing of alternative drugs but also a link to the respective dosing guidelines or an integrated dosage conversion tool. Furthermore, some of the participants stated that they would have liked the listed adverse drug events to be more specific.

“Toxicity is a very broad term. A lot of side effects that can be associated with it so maybe if there is a section that indicates specifically a tab like three most common toxicities associated with codeine use in ultrarapid metabolizers that would give me more a reassurance and confidence”–Pharmacy student 2.

A common issue amongst half of the interviewees was confusion and uncertainty about the two different guideline publishing consortia; especially the Dutch Pharmacogenetics Working Group (DPWG) (Swen et al., 2011) was so far unknown to them. One participant also commented that they wanted to know more about the origin and evidence of the guidelines, i.e., if they were derived from human or animal studies. However, in response to the question “Is there information you would like, but cannot find using the Safety Code system?” some participants also considered that it might be counter-productive to include additional information unless it is absolutely necessary and would not create the burden of excessive information.

“The more information you include, the more difficult it gets to weed through and tell what’s pertinent”–Clinical pharmacist 1.

#### General concerns

Five different concerns towards the implementation of a system like the MSC emerged from the analysis. Three participants expressed concerns regarding data privacy. Skepticism towards technology and possible change aversion of professionals were also mentioned. Some interviewees expressed doubts regarding the cost-benefit ratio of implementing such a system and one participant mentioned the potential negligence of other factors relevant for making a therapy decision as something he would be concerned about.

“The concern is there’s other factors besides just pharmacogenetics that dictate the kind of drug the patient should get, it’s not the only factor that determines what a patient should get … if they’ve gotten this before at a reasonable dose then it’s probably something that, and they didn’t experience any side effects then it might be something that they can keep using”–Pharmacy student 2.

### Adaptations

During the evaluation process, several issues and suggestions for improvement of the MSC Interface were identified. A list of all issues that led to changes of the interface during the three adaptation phases can be found in Table 6.

**Table 6:** Issues detected during the study and modifications made in response in the adaptation phases.

Phase	Issue	Adaptation in response to the issue
1	Confusion and uncertainty about displaying guidelines from two different consortia	The interface was split in two different versions: a “U.S. version” displaying the CPIC guidelines and a “European version” displaying the DPWG guidelines
2	Confusion about the headings and sections “critical” and “all”	The “all guidelines” list was removed so that the interface now displays only the critical drugs. The heading was changed from “critical” to “critical guidelines for this patient.”
3	Ambiguity whether the “last guideline update” date refers to the last MSC update or the update on sources (e.g. the latest version of CPIC guideline)	“Last guideline update” was changed to “date of evidence”

### Card layout evaluation

#### Ratings and comments

Table 7 shows the descriptive statistical measures for all five layouts split by professional group and in total. Overall, layout 1 and 5 received the highest and significantly better ratings than the other layouts (p = 0.02 and p = 0.003, respectively). A common point of critique regarding layout 1 was that the arrow system is too confusing and too difficult to follow. One participant also argued that with the arrow system prescribers might be tempted to adjust the dosage by themselves without looking up the recommended dosage. Out of all layouts, layout five had the highest number of positive comments, but also several concerns and suggestions for minor changes were expressed by the respondents. These included suggestions to replace the term “normal” in the bottom line with “wild-type” or “extensive metabolizer” and to add a statement that the drugs listed are not all inclusive. One participant felt that layout 5 might be confusing for some prescribers because the drugs are sorted by gene and therefore some drugs are listed in two different rows. Across all layouts, a common concern among participants was that the PGx information on the card (especially affected drugs) would become out of date soon and that the card would have to be reprinted frequently.

**Table 7:** Descriptive statistical measures of the card layout ratings by professional group and in total.

	Clinicians		Pharmacists		Researchers		Others		Overall	
	Median	IQR	Median	IQR	Median	IQR	Median	IQR	Median	IQR
**L1**	4	2	3	2	4	2	5	1	**4**	**1**
**L2**	4	1	2	2	3	2	3	1	**3**	**2**
**L3**	4	2	2.5	2	3	2	2	3	**3**	**2**
**L4**	4	2	2.5	2	1.5	3	1	1	**2**	**3**
**L5**	4	0	4	2	3	1	3	2	**4**	**1**

**Notes:**

L, Layout; IQR, Interquartile range.

#### Other information that should be printed on the card

The majority of participants confirmed that patients’ drug allergies based on medical history and laboratory contact details would be useful additional information that should be printed on the card (79.6% and 68.5%, respectively). The other possible selections “signature of laboratory head” and “patient signature” were less frequently chosen (13.0% and 16.7%, respectively). Other suggestions given through the free-text fields were: “primary care physician,” “nature of allergic reaction,” “social security number,” current medication list and “revision/update cycle.” Furthermore, one of the participants commented that information on drug allergies should not be printed on the card but should be captured in the QR code.

## Discussion

### Principal results

The main goal of this study was to evaluate and inform the further development of a mobile-based CDS system for PGx that can be deployed in various health care settings regardless of the availability of advanced and compatible CPOE systems. Overall, our results indicate that the MSC system is perceived positively by the majority of physicians and pharmacists across all investigated aspects (i.e., usability, trustworthiness, usefulness and feasibility of workflow integration). While we did not find any statistically significant differences in the attitudes towards the MSC system according to profession, awareness of and experience with CDS systems and genome-guided prescribing, this could have been fostered by the small scale of our study. The majority of participants were able to respond adequately to the patient scenarios indicating that the MSC interface presents information pertinent to decision-making in a user-friendly way. However, especially the second patient scenario generated a notable fraction of answers that are not in accordance with the recommendations made in the relevant guidelines. Based on participant comments, these can partly be explained by some physicians answering the question based on their clinical experience rather than solely on the displayed recommendation. Nevertheless, in other cases the reason remains unclear. The somewhat higher fraction of diverging answers in scenario 2 than in scenario 1 might point to a problem in the wording of the guideline itself. In view of the high positive predictive value of TPMT genotyping and the potentially life-threatening adverse drug events associated with the TPMT poor metabolizer phenotype, it is difficult to explain the number of PGx experts who decided to prescribe azathioprine at normal dosage in scenario 1. It is possible and common to measure TPMT enzyme activity directly in red blood cells and some expert groups such as the American College of Gastroenterology recommend TPMT phenotyping over genotyping to determine thiopurine drug dosages (Lichtenstein, Hanauer & Sandborn, 2009). This factor may have played a role in some participants’ choice, however, the specific reasons remain unclear.

During this study we furthermore identified several needs and suggestions for improvement of the MSC system, such as the confusion arising from displaying multiple and potentially differing recommendations from different guidelines. We pragmatically decided to split the UI into two versions to avoid confusion stemming from this issue in the subsequent evaluation phases. An informal review and comparison of overlapping DPWG and CPIC guidelines during the system’s development phase revealed slight discrepancies in the recommendations for certain CYP2C19 and CYP2D6 haplotypes and tricyclic antidepressants (e.g. “Initiate therapy with recommended starting dose” vs. “Reduce dose by 25% and monitor plasma concentration or select alternative drug” for CYP2D6*9/*9 and amitriptyline). For TPMT intermediate metabolizers and thiopurine drugs (in this example thiopurine), DPWG recommends “Select alternative drug.” while the CPIC recommendation says “Start with reduced doses (reduce by 30–50%) and adjust doses of thioguanine based on degree of myelosuppression and disease-specific guidelines.”). Other discrepancies observed were the frequent restriction to a specific target group in the CPIC guidelines (e.g. clopidogrel: only patients undergoing percutaneous coronary intervention). Ideally, only a single recommendation should be displayed, which—in case of the availability of multiple guidelines from different consortia—could also be based on a synthesis of available recommendations.

While one of the main advantages of the MSC system lies in its ability to implement PGx CDS in settings where adequate IT infrastructure such as CPOE systems and EHRs are lacking, the findings from our qualitative interviews emphasize the need for such a system to be able to communicate with or to be integrated into existing systems to accomplish optimal work flow incorporation in various health care settings. Furthermore, our study points out that a listing of alternative drugs could be helpful to save time in the drug prescribing process and consequently increase physicians’ and pharmacists’ confidence in using the system.

Additionally, our study revealed a wish for the guidelines to be more specific (e.g., state concrete drug dosages or include a dosage converting tool) among the participants. However, displaying concrete drug dosages adapted only to the patient’s PGx profile might also nourish an often mentioned concern: the negligence of other factors relevant for providing an optimal drug therapy, such as renal function or co-medication. Being able to include all of the relevant contextual factors for treatment would be the ideal situation but would once again require the existence of a sophisticated and mature health IT infrastructure—a requirement that is currently lacking in many European countries.

Other concerns that emerged from the interviews referred to pharmacogenetic testing in general: the unclear cost benefit-ratio and data privacy concerns. While positive influence on patient outcome as well as cost-effectiveness of pharmacogenetics-guided drug therapy has been demonstrated for several drug substances, the situation for other drugs indeed remains unclear or conflicting (Verhoef et al., 2015; Thompson et al., 2014; Lala et al., 2013; Olgiati et al., 2012). A preemptive pharmacogenetic testing approach could increase efficiency and reduce testing costs at the same time (Schildcrout et al., 2012).

Despite the Genetic Information Nondiscrimination Act (GINA) and the Health Insurance Portability and Accountability Act (HIPAA) and similar policies in European countries, concerns towards data privacy are still common when it comes to pharmacogenetic testing. Concerns that health insurance providers might get access to genetic test results are widespread and suggest a need for education about legal and regulatory backgrounds as well as about the significance of testing for variations in drug metabolism enzymes as opposed to testing for risk of disease (Tuteja et al., 2013).

Finally, the results of this study informed the development process of a prototypical pocket card that enables the widespread use PGx data and decision support across different health care settings. Our findings indicate that a gene-centered and tabulated presentation of the patient’s PGx profile on the pocket card is considered most helpful to call a physician’s or pharmacist’s attention to a patient being, e.g., a poor metabolizer and being in potential need for tailored therapy. Furthermore, our results emphasize the need for information transparency on the source of the PGx data (i.e., tested variants, laboratory contact details) to make such a system valuable for and accepted among PGx professionals.

### Related work

In recent years, there has been an increasing amount of literature on the development, implementation and evaluation of PGx CDS systems. Devine et al. (2014) evaluated the usability of PGx CDS alerts that were already embedded in a CPOE system among a small sample of cardiologists and oncologists. Lærum et al. (2014) developed a prototype for CYP3A5-based treatment during kidney transplantation and evaluated it among a small sample of hospital physicians. Both studies found that the physicians’ attitude towards clinical implementation of pharmacogenetics-based therapy by means of a CDS system was positive. Furthermore, both studies emphasized physicians’ preferences for seeing essential results and recommendations right away, with further explanations and references easily accessible but separated in order to prevent distraction from essential information needed for quick decision-making. In a recent study Overby et al. (2015) examined the impact of physicians characteristics (e.g., awareness and previous experience) on the communication effectiveness (i.e., changes in confidence in prescribing decisions, usefulness) of PGx alerts of a prototypical PGx CDS system embedded in an EHR. They did not find any association of previous experience with and awareness of PGx CDS systems with communication effectiveness of PGx alerts among the 22 included physicians. However, they reported a significant decrease of the physicians’ confidence in prescribing when presented with active and semi-active alerts generated by their embedded PGx CDS prototype as compared to viewing information on genetic variants prior to ordering a medication. The present study builds on and complements the existing literature by providing insights into the perceptions of an alternative, mobile-based and thus highly flexible way of providing concise PGx CDS that can be deployed in various healthcare settings independent of existing EHRs and CPOEs. Furthermore, while much of the available literature on PGx CDS focuses on how alert messages should be structured and presented, this is the first study to explore ways to alert health care providers without advanced PGx knowledge in clinical and outpatient settings to the availability of essential PGx therapy modifications by means of a personalized pocket card. These findings, furthermore, complement existing research that focuses on patient preferences regarding the storage of PGx test results and on the development of patient-friendly genomic test reports to ensure lifelong benefits of PGx testing (Haga et al., 2011; Haga et al., 2012).

### Limitations

A limitation of the study lies in the modest survey response rate of Survey B. This might have led to higher evaluation scores of the MSC system in this part of the study due to the assumable higher fraction of technology enthusiasts among the participating physicians and pharmacists. Furthermore, the study was limited by the fact that the interview transcripts were coded by only one researcher without establishing inter-rater reliability. Coding by more than one researcher would possibly have led to slightly different categorizations. However, it is unlikely that this limitation has substantially and negatively influenced the overall study goal since the aim of the qualitative interviews was to identify broad tendencies rather than detailed lists of concerns. Finally, due to the transnational approach of our study, participants were recruited from very heterogeneous health care systems across and within the different phases of this study. The vast majority of PGx experts who participated in Survey A were from the USA. This may be explained by the fact that the clinical implementation of PGx is currently far more common and advanced in the USA resulting in a higher number of eligible participants as compared to most European countries. While this limitation resulted in an underrepresentation of European viewpoints on the card layout, the evaluation may have benefitted from the US participants’ advanced experience in the actual clinical implementation of PGx. The transnational aspect of this study might also have influenced the evaluation of the MSC system itself, especially regarding workflow integration. However, this limitation is at least partly mitigated due to the MSC system’s independence of existing health IT infrastructure.

## Conclusions

This is the first study to examine attitudes towards the usefulness and usability of a flexible mobile-based CDS system for pharmacogenetics-guided drug therapy that can be easily integrated into existing care processes and infrastructures. Our study captures a breadth of viewpoints ranging from PGx experts from various disciplines to physicians and pharmacists without advanced PGx knowledge. Our mixed methods approach allowed for a comprehensive and complementary evaluation of the MSC system and provided a transnational perspective. Our findings suggest that the very concise presentation of the essential facts and recommendations by the MSC interface is deemed acceptable for guiding clinical decisions and that the system is perceived positively by the physicians and pharmacists included in the study. Our findings also point out that including a list of alternative drugs could help to increase user acceptance of PGx CDS systems. Furthermore, this study provides key insights into how human-readable PGx information can be used to alert health professionals to the availability of essential therapy modifications for a specific patient. A gene-centered and tabulated presentation of the patient’s PGx profile along with a listing of critically affected drugs is deemed most useful by professionals. The results of this study will inform the further evolution of the MSC system. Finally, our findings suggest that adequate education about legal and regulatory backgrounds regarding the use of pharmacogenetic information as well as information about the cost-benefit ratio of pharmacogenetic testing will be necessary to achieve optimal user acceptance of PGx CDS.

## Supplemental Information

10.7717/peerj.1671/supp-1 Supplemental Information 1 Additional file 1: Information leaflet.

10.7717/peerj.1671/supp-2 Supplemental Information 2 Additional file 2: Interview guide.

10.7717/peerj.1671/supp-3 Supplemental Information 3 MSC card layout survey responses.

10.7717/peerj.1671/supp-4 Supplemental Information 4 MSC usability survey responses.

## List of abbreviations

CDS: Clinical Decision Support
CPIC: Clinical Pharmacogenetics Implementation Consortium
CPOE: Computerized Provider Order Entry
DPWG: Dutch Pharmacogenetics Working Group
EHR: Electronic Health Record
MSC: Medication Safety Code
PGx: Pharmacogenomics
QR: Quick Response
SUS: System Usability Scale
UI: User Interface

## Additional Information and Declarations

### Data Deposition

The following information was supplied regarding data availability: Anonymized raw data are provided as Supplementary Files. Raw interview transcripts are not published for privacy reasons.
