# Supplementary material for: Examining perceptions of the usefulness and usability of a mobile-based system for pharmacogenomics clinical decision support: a mixed methods study
Source: PeerJ. 2016 Feb 8;4:e1671. doi: 10.7717/peerj.1671 (PMC4768706; doi:10.7717/peerj.1671)
Supplement: Supplemental Information 1 [file peerj-04-1671-s001.pdf]

The Safety Code Initiative

Putting personalized medicine into the pockets of patients

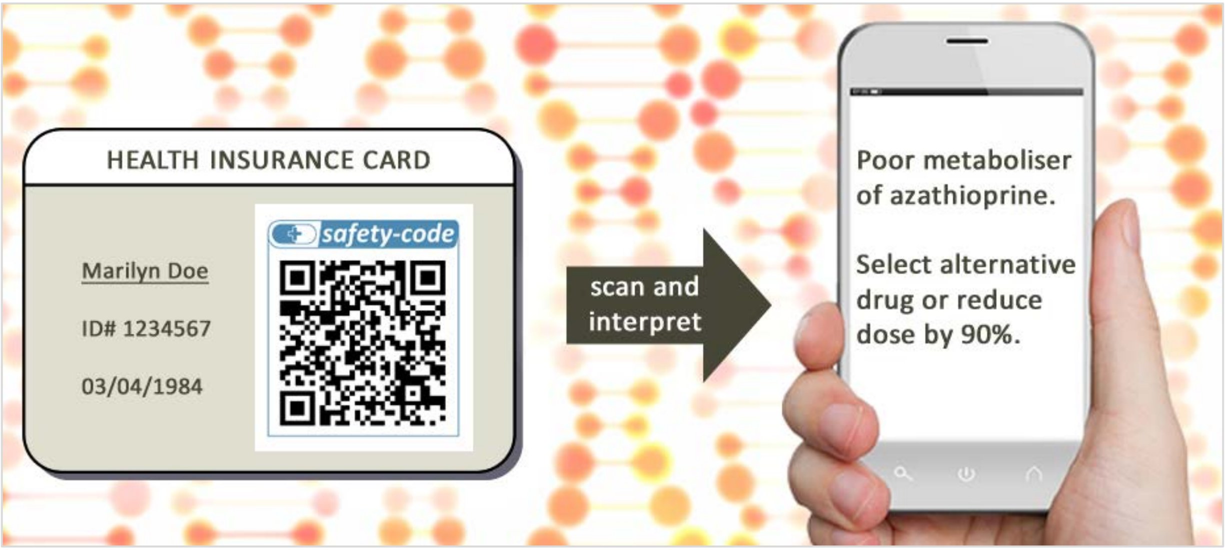

Why you should care

The efficacy and safety of medications for individual patients can be better predicted when data about essential *pharmacogenes* of patients are known. Pharmacogenes are genes that are responsible for the metabolism, transport and effects of many common medications.

The goal of the Safety Code initiative is to make data and clinical guidelines about these pharmacogene sand other safety-critical patient traits easily available for all patients in all health care settings.

How it works

1) A patient carries their Safety Code with them and hands it to a health care professional when asked for it.
2) When prescribing a new medication or modifying existing prescriptions, the health care professional can scan the Safety Code with a common smartphone and see recommendations for drug dosing and drug choice based on the individual pharmacogenomic markers of the patient.

Quick Facts

→ **Essential pharmacogenomic data of a patient are captured in a two-dimensional barcode (‘QR code’).** Apps for reading QR codes come pre-installed on most devices (e.g., ‘Google Goggles’ on Android phones), or can easily be installed from app stores. The QR code leads to a web page that gives recommendations based on up-to-date clinical guidelines.

→ **Privacy aware: All data are inside the QR code and remain anonymous.** No central database is required; patients have full control over their data just like they have control over the contents of their wallets. Optionally, client-side decoding without web access is possible.

→ The system currently contains **400 guidelines** for more than **60 pharmaceutical compounds** based on **60 pharmacogenes**. The system is based on guidelines issued by the committees of the *Clinical Pharmacogenetics Implementation Consortium* and the *Dutch Pharmacogenetics Working Group*, the leading international groups in clinical pharmacogenomics.

→ **The system is openly available and backed by an international team of experts.**

Visit http://safety-code.org to find out more

Contact: Dr. Matthias Samwald, Medical University of Vienna, matthias.samwald@meduniwien.ac.at

MEDICAL UNIVERSITY OF VIENNA · safety-code The Medicine Safety Code initiative · University of Pittsburgh
