# Supplementary material for: Examining perceptions of the usefulness and usability of a mobile-based system for pharmacogenomics clinical decision support: a mixed methods study
Source: PeerJ. 2016 Feb 8;4:e1671. doi: 10.7717/peerj.1671 (PMC4768706; doi:10.7717/peerj.1671)
Supplement: Supplemental Information 2 [file peerj-04-1671-s002.pdf]

a. Assuming that you had the technology necessary, and your patients had genotyping results in the right format, does the Safety Code system fit into your workflow?

b. What are the barriers to using the Safety Code system in your current workflow?

c. What do you like about the Safety Code system?

d. What are your concerns about the Safety Code system?

e. What do you like about how the Safety Code system presents information?

f. What do you dislike about how the Safety Code system presents information?

g. Is there information you would like, but cannot find using the Safety Code system?

h. Does the Safety Code system provide you with the information that you need to feel comfortable in making a recommendation about codeine and Marilyn?

i. Do you have any other comments about the Safety Code system?
